# A Note on Complexities by Means of Quantum Compound Systems

**DOI:** 10.3390/e22030298

**Published:** 2020-03-05

**Authors:** Noboru Watanabe

**Affiliations:** Department of Information Sciences, Tokyo University of Science, Noda City, Chiba 278-8510, Japan; watanabe@is.noda.tus.ac.jp

**Keywords:** quantum entropy, quantum information, quantum compound system

## Abstract

It has been shown that joint probability distributions of quantum systems generally do not exist, and the key to solving this concern is the compound state invented by Ohya. The Ohya compound state constructed by the Schatten decomposition (i.e., one-dimensional orthogonal projection) of the input state shows the correlation between the states of the input and output systems. In 1983, Ohya formulated the quantum mutual entropy by applying this compound state. Since this mutual entropy satisfies the fundamental inequality, one may say that it represents the amount of information correctly transmitted from the input system through the channel to the output system, and it may play an important role in discussing the efficiency of information transfer in quantum systems. Since the Ohya compound state is separable state, it is important that we must look more carefully into the entangled compound state. This paper is intended as an investigation of the construction of the entangled compound state, and the hybrid entangled compound state is introduced. The purpose of this paper is to consider the validity of the compound states constructing the quantum mutual entropy type complexity. It seems reasonable to suppose that the quantum mutual entropy type complexity defined by using the entangled compound state is not useful to discuss the efficiency of information transmission from the initial system to the final system.

## 1. Introduction

The first scholar to give much attention to a mathematical treatment of communication processes was Shannon [1]. He created the information theory by introducing measures of information, such as the entropy of the system and the mutual entropy formulated by the relative entropy of the joint probability distribution between input and output determined by the channel and the direct product distribution between input and output. Various researchers have studied the efficiency of information transmission from the input system to the output system passing through ordinary communication channels based on information theory. To rigorously examine the efficiency of information transmission in optical communication, it is necessary to formulate quantum information theory that can describe such quantum effects. It is indispensable to extend important measures, such as entropy, to quantum systems and greatly expand them to more general information theories, including Shannon’s information theory.

A study to extend entropy to quantum systems was started by von Neumann [2] in 1932. Furthermore, the quantum relative entropy was introduced by Umegaki [3], and Araki [4,5], Uhlmann [6], Donald [7] extended it to more general quantum systems. One of the important problem is to examine how accurately information is transmitted when an optical signal is passed through an optical channel. To achieve this, it needs to extend the mutual entropy determined in the classical system to the quantum system.

The mutual entropy of a classical system is determined using the joint probability distribution between the input and the output systems. However, it has been shown that the joint probability distribution of the quantum system generally does not exist [8]. Ohya [9,10] introduced the compound state (Ohya compound state) representing correlation between the initial state and the output state to construct the quantum mutual entropy in quantum communication processes. Ohya formulated the quantum mutual entropy [9,10,11,12,13,14,15] by using the quantum relative entropy between the Ohya compound state and the tensor product of the input state and the output state through the quantum channel. Then the Shannon’s type inequalities hold [9,10]. It was extended to C*-algebra by Ohya [12]. Based on the Ohya mutual entropy, the quantum capacity has been studied by several researchers [16,17,18,19,20,21]. Added to these entropies, Ohya defined the C*-mixing entropy [22] and it was extended for the Rényi case [23]. The property of these entropies was study in [11,13,23,24]. The entangled state is an important subject for studying quantum information theory. One of the noticeable results to discuss the entanglement state is the Jamiołkowski’s isomorphism [25].

The purpose of this paper is to consider the validity of the compound states constructing the quantum mutual entropy type complexity. In this paper, we show the construction of the Ohya compound state by using the Jamiolkowski isomorphism, and we review the existence of completely positive channel between the entangled compound state and the Ohya compound state. We introduce the mutual entropy type measure by using the quantum relative entropy between the hybrid entangled compound state and trivial compound state, and study some property of the mutual entropy type measure with respect to the hybrid entangled compound state. The important applications of the entangled quantum channels are the quantum teleportation and the quantum dense coding, and so on. To investigate the efficiency of information transmission of these entangled quantum channels [26], it is debatable whether the mutual entropy type complexity by using the entangled compound state [26] is useful. Since the quantum teleportation can be described by the completely positive channel, it is also used in this paper the same as the usual quantum information. We show the quantum mutual entropy type measure defined by using the entangled compound state is not suitable to discuss the efficiency of information transmission from the initial system to the final system.

## 2. Quantum Entropy for Density Operators

Von Neumann defined the entropy of a quantum state ρ by

S(ρ)=−trρlogρ.

The Schatten decomposition of a state ρ is described by

$$\rho =\sum_{n}\lambda_{n}E_{n},$$

where $\lambda_{n}$ is an eigenvalue of ρ and $E_{n}$ is the one-dimensional projection with respect to $\lambda_{n}$. This Schatten decomposition is not unique excepting all eigenvalues are non-degenerate. For a state ρ, the von Neumann entropy is equal to the Shannon entropy with respect to the probability distribution $\left\{\lambda_{n}\right\}$:

$$S\left(\rho \right)=-\sum_{n}\lambda_{n}\log\lambda_{n}.$$

Hence the von Neumann entropy includes the Shannon entropy as a special case.

## 3. Quantum Channels

Let $A_{1}$ $\left(\mathrm{resp}.\;A_{2}\right)$ be a C${}^{*}$-algebra or $B(H_{1})$ (resp. $B(H_{1})$) the set of all bounded operators on a separable complex Hilbert space $H_{1}$ $\left(\mathrm{resp}.\;H_{2}\right).$ We denote the input (resp. output) quantum system by $S(A_{1})$ $\left(\mathrm{resp}.\;S(A_{2})\right).$ ($A_{1}$, $S(A_{1})$) (resp. $(A_{2}$, $S(A_{2}))$ is the input (resp. output) quantum system. Let Λ is a linear mapping from $A_{2}$ to $A_{1}$ with $\Lambda \left(I_{2}\right)=I_{1}$, where $I_{k}$ is the identity operator in $A_{k}$ (k=1,2). The dual map $\Lambda^{*}$ of Λ is a linear quantum channel from $S(A_{1})$ to $S(A_{1})$ given by $\Lambda^{*}\left(\varphi \right)\left(B\right)=\varphi \left(\Lambda (B)\right)$ for any $\varphi \in S(A_{1})$ and any $B\in A_{2}$. If Λ holds

$$\sum_{i,j=1}^{n}A_{i}^{*}\Lambda \left(B_{i}^{*}B_{j}\right)A_{j}\geq 0$$

for all n∈N, all $B_{j}\in A_{2}$ and all $A_{j}\in A_{1}$ is said to be a completely positive (C.P.) channel [11,13,14,15,27,28].

### 3.1. Quantum Communication Processes

$K_{1}$ and $K_{2}$ are two Hilbert spaces representing noise and loss systems. Let $S\left(H_{k}\right)$ (resp. $S\left(K_{k}\right)$) be the set of all density operators on $H_{k}$ (resp. $K_{k}$) (k=1,2). Put $A_{1}=B\left(H_{1}\right)$, $A_{2}=B\left(H_{2}\right)$, $S\left(A_{1}\right)=S\left(H_{1}\right),$$S\left(A_{2}\right)=S\left(H_{2}\right)$, $B_{2}=B\left(K_{1}\right),$$B_{2}=B\left(K_{2}\right)$, $S\left(B_{1}\right)=S\left(K_{1}\right)$ and $S\left(B_{2}\right)=S\left(K_{2}\right)$.

Ohya [9] formulated a model of quantum channel with respect to quantum communication process considering noise and loss.

Let $\gamma^{*}$ be a CP channel from $S\left(A_{1}\right)$ to $S\left(A_{1}⊗B_{1}\right)$ defined by

$$\gamma^{*}\left(\varphi \right)=\varphi ⊗\psi$$

for any input state $\varphi \in S(A_{1})$ and any noise state $\psi \in S\left(B_{1}\right)$ and $a^{*}$ be a CP channel from $S\left(A_{2}⊗B_{2}\right)$ to $S\left(A_{2}\right)$ given by

$$a^{*}\left(\Psi \right)\left(A\right)=\Psi \left(A⊗I_{2}\right)$$

for any $\Psi \in S\left(A_{2}⊗B_{2}\right)$ and any $A\in A_{2}$, where $I_{k}$ is the identity operator in $B_{k}$ (k=1,2). $\pi^{*}$ is a CP channel from $S\left(A_{1}⊗B_{1}\right)$ to $S\left(A_{2}⊗B_{2}\right)$ depending on the physical properties of the communication device. For all input state $\varphi \in S(A_{1})$ and all $A\in A_{2}$, he quantum channel $\Lambda^{*}$ with respect to the communication process is defined by

$$\begin{array}{ccc} \Lambda^{*}\left(\varphi \right)\left(A\right) & \equiv & \pi^{*}\left(\;\varphi ⊗\psi \right)\left(A⊗I_{2}\right)\\ & = & \left(a^{*}\circ \pi^{*}\circ \gamma^{*}\right)\left(\varphi \right)\left(A\right). \end{array}$$

We here briefly review the noisy optical channel and the attenuation channel in respect of the quantum communication processes

### 3.2. Noisy Quantum Channel

Let ψ be a normal state in $S\left(B_{1}\right)$ and $\tilde{\psi }=|m_{1}\rangle \langle m_{1}|\in S\left(K_{1}\right)$ be the $m_{1}$ photon number state satisfying $\psi \left(B\right)=tr\tilde{\psi }B=\langle m_{1},Bm_{1}\rangle$ for any $B\in B_{1}$. Let *V* be a linear mapping from $H_{1}⊗K_{1}$ to $H_{2}⊗K_{2}$ given by

$$V\left(|n_{1}\rangle ⊗|m_{1}\rangle \right)=\sum_{j=0}^{n_{1}+m_{1}}C_{j}^{n_{1},m_{1}}|j\rangle ⊗|n_{1}+m_{1}-j\rangle ,$$

$$\begin{array}{ccc} C_{j}^{n_{1},m_{1}} & = & \sum_{r=L}^{K}{\left(-1\right)}^{n_{1}+j-r}\frac{\sqrt{n_{1}!m_{1}!j!\left(n_{1}+m_{1}-j\right)!}}{r!\left(n_{1}-j\right)!\left(j-r\right)!\left(m_{1}-j+r\right)!}\\ & & \times \alpha^{m_{1}-j+2r}{\left(-\bar{\beta }\right)}^{n_{1}+j-2r}, \end{array}$$

where $|n_{1}\rangle$ is the $n_{1}$ photon number state vector in $H_{1}$, and α, β are complex numbers holding ${\left|\alpha \right|}^{2}+{\left|\beta \right|}^{2}=1$, $\;K=\min\{n_{1},j\}$ and $L=\max\{m_{1}-j,0\}$. For all $A\in A_{2}$, we introduced the noisy optical channel $\Lambda^{*}$ [19] with a normal state ψ by

$$\begin{array}{ccc} \Lambda^{*}\left(\varphi \right)\left(A\right) & \equiv & \pi^{*}\left(\varphi ⊗\psi \right)\left(A⊗I_{2}\right)\\ & = & \left(\varphi ⊗\psi \right)\circ \pi \left(A⊗I_{2}\right)\\ & = & trV\left(\tilde{\varphi }⊗\tilde{\psi }\right)V^{*}\left(A⊗I_{2}\right) \end{array}$$

where $\tilde{\varphi }\in S\left(H_{1}\right)$ be the density operator holding $\varphi \left(A\right)=tr\tilde{\varphi }A$ for any $A\in A_{1}$. 

The noisy quantum channel defined on the input, noise, output and loss systems generated by all photon number states of each system deals with the optical noise state created by the photon number states. The noisy quantum channel contains the generalized beam splitter transmitting from the input and noise systems to the output and loss systems. We have the following theorem [29].

**Theorem** **1.** The noisy optical channel $\Lambda^{*}$ with noise state

$$\psi \left(B\right)=tr\left[|m\rangle \langle m|B\right]\;\left(B\in B\right)$$

*is described by*

(1) $$\Lambda^{*}\left(\varphi \right)\left(A\right)=tr\left[\sum_{i=0}^{\infty }O_{i}VQ^{\left(m\right)}\tilde{\varphi }Q^{\left(m\right)*}V^{*}O_{i}^{*}A\right],\left(A\in \bar{A}\right)$$

*where $Q^{\left(m\right)}\equiv \sum_{l=0}^{\infty }\left(|y_{l}\rangle ⊗|m\rangle \right)\langle y_{l}|,$$O_{i}\equiv \sum_{k=0}^{\infty }|z_{k}\rangle \left(\langle z_{k}|⊗\langle i|\right)$, $\left\{|y_{l}\rangle \right\}$ is a CONS in $H_{1}$, $\left\{|z_{k}\rangle \right\}$ is a CONS in $H_{2}$ and $\left\{|i\rangle \right\}$ is the set of number states in $K_{2}$.*

$\pi^{*}$ [19] is said to be a generalized beam splitting. For the coherent input state $\Phi_{\xi ⊗\kappa }\left(\tilde{A}\right)$, the output state of $\pi^{*}$ is obtained by

$$\pi^{*}\left(\Phi_{\xi ⊗\kappa }\right)\left(\tilde{A}\right)=\Phi_{\alpha \xi +\beta \kappa ⊗-\bar{\beta }\xi +\bar{\alpha }\kappa }\left(\tilde{A}\right)\;\left(\tilde{A}\in A_{1}⊗A_{2}\right)$$

The attenuation channel [9] is the noisy optical channel with a vacuum noise.

### 3.3. Attenuation Channel

Let $\psi_{0}$ be a normal state in $S\left(B_{1}\right)$ and ${\tilde{\psi }}_{0}=|0\rangle \langle 0|\in S\left(K_{1}\right)$ be the vacuum noise state satisfying $\psi_{0}\left(B\right)=tr{\tilde{\psi }}_{0}B=\langle 0,B0\rangle$ for any $B\in B_{1}$. Let *V* be a linear mapping from $H_{1}⊗K_{1}$ to $H_{2}⊗K_{2}$ given by

$$\begin{array}{ccc} V_{0}\left(|n_{1}\rangle ⊗|0\rangle \right) & = & \sum_{j}^{n_{1}}C_{j}^{n_{1}}|j\rangle ⊗|n_{1}-j\rangle ,\\ C_{j}^{n_{1}} & = & \sqrt{\frac{n_{1}!}{j!(n_{1}-j)!}}\alpha^{j}{\left(-\bar{\beta }\right)}^{n_{1}-j} \end{array}$$

where $|n_{1}\rangle$ is the $n_{1}$ photon number state vector in $H_{1}$, and α, β are complex numbers holding ${\left|\alpha \right|}^{2}+{\left|\beta \right|}^{2}=1$. For any $A\in A_{2}$, the attenuation channel $\Lambda^{*}$ [9] with a vacuum noise state $\psi_{0}$ is given by

$$\begin{array}{ccc} \Lambda_{0}^{*}\left(\varphi \right)\left(A\right) & \equiv & \pi_{0}^{*}\left(\varphi ⊗\psi_{0}\right)\left(A⊗I_{2}\right)\\ & = & \left(\varphi ⊗\psi_{0}\right)\circ \pi_{0}\left(A⊗I_{2}\right)\\ & = & trV_{0}\left(\tilde{\varphi }⊗{\tilde{\psi }}_{0}\right)V_{0}^{*}\left(A⊗I_{2}\right). \end{array}$$

It represents the beam splitting sending the input state to the output and loss states, which can be described as the transformation process from the tensor product of the input state and the vacuum noise state to the tensor product of the output and loss states. Let $E_{0}^{*}$ be a lifting from $S\left(H\right)$ to $S\left(H⊗K\right)$ [30]. The beam splitting [31] is defined on generalized Fock spaces by

$$E_{0}^{*}\left(|\xi \rangle \langle \xi |\right)=|\alpha \xi \rangle \langle \alpha \xi |⊗|\beta \xi \rangle \langle \beta \xi |$$

The entangled quantum channels are the quantum teleportation and the quantum dense coding, and it is discussed in this paper as the completely positive channel.

## 4. Quantum Mutual Entropy

For purely quantum systems, the mutual entropy $I\left(\rho ;\Lambda^{*}\right)$ in respect of an input quantum state ρ and a quantum channel $\Lambda^{*}$ needs to satisfy the following conditions: (i) the identity channel $\Lambda^{*}=id$, the quantum mutual entropy is equal to the von Neumann entropy of ρ (i.e., $I\left(\rho ;id\right)$$=S\left(\rho \right))$. (ii) For the classical systems, the quantum mutual entropy agrees to classical mutual entropy. (iii) Shannon’s type fundamental inequalities 0≤$I\left(\rho ;\Lambda^{*}\right)\leq S\left(\rho \right)$ is satisfied.

For the Schatten decomposition $\sum_{n}\lambda_{n}E_{n}$ [32] of the input state ρ and the quantum channel $\Lambda^{*}$, Ohya proposed the compound state $\sigma_{E}$ defined by

$$\sigma_{E}=\sum_{n}\lambda_{n}E_{n}⊗\Lambda^{*}E_{n}.$$

For the compound states $\sigma_{E}$ and $\sigma_{0}=\rho ⊗\Lambda^{*}\rho$, Ohya [9,10] defined the quantum mutual entropy (information) by taking the Umegaki’s relative entropy [3] $S\left(\sigma_{E},\sigma_{0}\right)$ over all Schatten decompositions of ρ

$$I\left(\rho ;\Lambda^{*}\right)=\sup\left\{S\left(\sigma_{E},\sigma_{0}\right);E=\left\{E_{n}\right\}\right\},$$

where $S\left(\sigma_{E},\sigma_{0}\right)$ is given by

$$S\left(\sigma_{E},\sigma_{0}\right)=\left\{\begin{array}{cc} tr\sigma_{E}\left(\log\sigma_{E}-\log\sigma_{0}\right) & \left(s\left(\sigma_{E}\right)\ll s\left(\sigma_{0}\right)\right),\\ \infty & \left(\mathrm{else}\right), \end{array}\right.$$

$s\left(\sigma_{E}\right)\ll s\left(\sigma_{0}\right)$ indicates that the support projection $s\left(\sigma_{0}\right)$ of $\sigma_{0}$ is larger than the support projection $s\left(\sigma_{E}\right)$ of $\sigma_{E}$. The quantum mutual entropy satisfies the above conditions (1) ∼ (3) [9]:

**Theorem** **2.** $$0\leq I\left(\rho ;\Lambda^{*}\right)\leq \min\left\{S\left(\rho \right),S\left(\Lambda^{*}\rho \right)\right\}.$$

For a linear channel, one has the following form [9]:

**Theorem** **3.** *The quantum mutual entropy is denoted as*

$$I\left(\rho ;\Lambda^{*}\right)=\sup\left\{\sum_{n}\lambda_{n}S\left(\Lambda^{*}E_{n},\Lambda^{*}\rho \right);E=\left\{E_{n}\right\}\right\}.$$

When the input system reduces to classical one, an input state ρ is represented by a probability distribution or a probability measure. Then the Schatten decomposition of ρ is unique, namely for the case of probability distribution; $\rho =\left\{\mu_{k}\right\},$

$$\rho =\sum_{k}\mu_{k}\delta_{k},$$

where $\delta_{k}$ is the Dirac delta measure, the mutual entropy is described by

$$I\left(\rho ;\Lambda^{*}\right)=\sum_{k}\mu_{k}S\left(\Lambda^{*}\delta_{k},\Lambda^{*}\rho \right),$$

which is equal to

$$I\left(\rho ;\Lambda^{*}\right)=S\left(\Lambda^{*}\rho \right)-\sum_{k}\mu_{k}S\left(\Lambda^{*}\delta_{k}\right).$$

This equation introduced by Levitin [33] and Holevo [34] associated with classical-quantum channels. The classical-quantum channel is called the quantum coding (see [13,14,15]). This equation has no meaning unless one of the two terms is finite for an infinite-dimensional Hilbert space. The Ohya mutual entropy contains their semi-classical mutual entropies as a special case.

For a completely positive (CP) channel $\Lambda^{*}$, it can be represented by

$$\Lambda^{*}\left(\rho \right)=\sum_{n}V_{n}\rho V_{n}^{*}$$

where $\sum_{n}V_{n}^{*}V_{n}=I$ is held. The compound state is constructed by using the compound lifting $E^{*}$ associated with a fixed decomposition of ρ as ρ$=\sum_{n}\mu_{n}\rho_{n}$ ($\rho_{n}\in S\left(A_{1}\right)$) such as

$$E^{*}\rho =\sum_{n}\mu_{n}\rho_{n}⊗\Lambda^{*}\rho_{n}.$$

## 5. Entropy Exchange and Coherent Information

For a completely positive channel $\Lambda^{*}$ denoted by

$$\Lambda^{*}\left(\rho \right)=\sum_{i}V_{i}\rho V_{i}^{*},\;\sum_{i}V_{i}^{*}V_{i}=1,$$

the *entropy exchange [35,36,37,38,39]* of the quantum operation $\Lambda^{*}$ and the input state ρ is given by

$$\begin{array}{ccc} S_{e}\left(\rho ,\Lambda^{*}\right) & = & S(W)=-\mathrm{tr}W\log W,\\ W & = & \left(w_{ij}\right)=\left(\mathrm{tr}V_{i}\rho V_{j}^{*}\right), \end{array}$$

where $w_{ij}$ is the matrix elements of *W*. The coherent entropy [39] and the Lindblad–Neilsen entropy [35] are defined as follows:

**Definition** **1.** *The coherent entropy is defined by*

$$I_{c}\left(\rho ,\Lambda^{*}\right)=S\left(\Lambda^{*}\rho \right)-S_{e}\left(\rho ,\Lambda^{*}\right)$$

*The Lindblad–Neilsen entropy is defined by this coherent entropy with the von Neumann entropy S(ρ) such that*

$$I_{LN}\left(\rho ,\Lambda^{*}\right)\equiv S\left(\rho \right)+S\left(\Lambda^{*}\rho \right)-S_{e}\left(\rho ,\Lambda^{*}\right)$$

The coherent entropy was defined by reducing the von Neumann entropy of the output state by the entropy exchange. It can be used for the efficient channel of the physical systems. The Lindblad-Nielsen entropy was defined by adding the coherent entropy to the von Neumann entropy of the input state. It seems that it can be used to explain the quantum dense coding in the quantum information. It should also be added that the quantum mean mutual entropy [24] and quantum dynamical mutual entropy [40,41] are discussed. Those mutual entropy type complexities satisfy the Shannon’s type fundamental inequalities.

## 6. Comparison of Various Quantum Mutual Type Entropies

Based on [14,15], we briefly show the comparison of these mutual entropy type complexities.

Let $\left\{x_{n}\right\}$ be a CONS in the input Hilbert space $H_{1}$ and $A_{n}=|x_{n}\rangle \langle x_{n}|$ be a one-dimensional projection holding

$$\sum_{n}A_{n}=I.$$

For the quantum channel $\Lambda^{*}$ denoted by

$$\Lambda^{*}\left(•\right)\equiv \sum_{n}A_{n}•A_{n},$$

we have the following theorems [14,15]:

**Theorem** **4.** When $\left\{A_{j}\right\}$ is a projection valued measure and dim(ran$A_{j})=1,$ for arbitrary state ρ we have (1) $I\left(\rho ,\Lambda^{*}\right)\leq \min\left\{S\left(\rho \right),S\left(\Lambda^{*}\rho \right)\right\}$, (2) $I_{C}\left(\rho ,\Lambda^{*}\right)=0,$ (3) $I_{LN}\left(\rho ,\Lambda^{*}\right)=S\left(\rho \right).$

**Theorem** **5.** Let in the input Hilbert space be given a CONS $\left\{x_{n}\right\}$ and in the output Hilbert space a sequence of the density operators $\left\{\rho_{n}\right\}$. Consider a channel $\Lambda^{*}$ given by

$$\Lambda^{*}\left(\rho \right)=\sum_{n}\langle x_{n}|\rho |x_{n}\rangle \;\rho_{n}$$

*where ρ is any state in the input Hilbert space. Then the coherent entropy is equals to 0 for any state ρ.*

For the attenuation channel $\Lambda_{0}^{*}$, the following theorems are held [14,42]:

**Theorem** **6.** For any state $\rho =\sum_{n}\lambda_{n}|n\rangle \langle n|$ and the attenuation channel $\Lambda_{0}^{*}$ with ${\left|\alpha \right|}^{2}={\left|\beta \right|}^{2}=\frac{1}{2}$, one has

*1.*  $0\leq I\left(\rho ;\Lambda_{0}^{*}\right)\leq \min\left\{S\left(\rho \right),S\left(\Lambda_{0}^{*}\rho \right)\right\}$ (Ohya mutual entropy),
*2.*  $I_{C}\left(\rho ;\Lambda_{0}^{*}\right)=0$ (coherent entropy),
*3.*  $I_{LN}\left(\rho ;\Lambda_{0}^{*}\right)=S\left(\rho \right)$ (Lindblad-Nielsen entropy).

**Theorem** **7.** For the attenuation channel $\Lambda_{0}^{*}$ and the input state $\rho =\lambda |0\rangle \langle 0|+\left(1-\lambda \right)|\theta \rangle \langle \theta |$, we have

*1.*  $0\leq I\left(\rho ;\Lambda_{0}^{*}\right)\leq \min\left\{\;S\left(\rho \right),S\left(\Lambda_{0}^{*}\rho \right)\right\}$ (Ohya mutual entropy),
*2.*  $-S\left(\rho \right)\leq I_{C}\left(\rho ;\Lambda_{0}^{*}\right)\leq S\left(\rho \right)$ (coherent entropy),
*3.*  $0\leq I_{LN}\left(\rho ;\Lambda_{0}^{*}\right)\leq 2S\left(\rho \right)$ (Lindblad-Nielsen entropy).

The above theorem means that for ${\left|\alpha \right|}^{2}<{\left|\beta \right|}^{2}$, the coherent entropy $I_{C}\left(\rho ;\Lambda_{0}^{*}\right)$ less than 0 and for ${\left|\alpha \right|}^{2}>{\left|\beta \right|}^{2}$, the Lindblad-Nielsen entropy $I_{LN}\left(\rho ;\Lambda_{0}^{*}\right)$ is greater than the von Neumann entropy $S\left(\rho \right)$.

From what has been obtained above, we may, therefore, reasonably conclude that Ohya mutual entropy $I\left(\rho ;\Lambda^{*}\right)$ only satisfies the inequality held in classical systems, so that Ohya mutual entropy may be the best candidate as a quantum extension of classical mutual entropy. The main reason is that the Ohya mutual entropy holds the above three conditions in Section 4. The coherent entropy does not satisfy (iii) and the Lindblad–Nielsen entropy does not satisfy (i) and (iii).

The noisy optical channel $\Lambda^{*}$ can be described by using the Stinespring–Sudarshan–Kraus form.

**Theorem** **8.** The noisy optical channel $\Lambda^{*}$ with noise state $|m\rangle \langle m|$ is described by

$$\Lambda^{*}\left(\rho \right)=\sum_{i=0}^{\infty }O_{i}VQ^{\left(\gamma \right)}\rho Q^{\left(\gamma \right)*}V^{*}O_{i}^{*},$$

*where $Q^{\left(m\right)}\equiv \sum_{l=0}^{\infty }\left(|y_{l}\rangle ⊗|\gamma \rangle \right)\langle y_{l}|,$$O_{i}\equiv \sum_{k=0}^{\infty }|z_{k}\rangle \left(\langle z_{k}|⊗\langle i|\right)$, $\left\{|y_{l}\rangle \right\}$ and $\left\{|z_{k}\rangle \right\}$ are CONS in $H_{1}$ and $H_{2}$, respectively. $\left\{|i\rangle \right\}$ is the set of number states in $K_{2}$.*

**Theorem** **9.** For the noisy optical channel $\Lambda^{*}$ with α,β satisfying ${\left|\alpha \right|}^{2}+{\left|\beta \right|}^{2}=1$ and the input state $\rho =\lambda |0\rangle \langle 0|+\left(1-\lambda \right)|\theta \rangle \langle \theta |$, we have the entropy exchange $S\left(W\right),$$W=\left(w_{ij}\right),$$w_{ij}=trW_{i}\rho W_{j}^{*},$$W_{i}=O_{i}VQ^{\left(\gamma \right)}$

$$\begin{array}{ccc} w_{ij} & = & trW_{i}\rho W_{j}^{*}\\ & = & \sum_{n}\langle x_{n},W_{i}\rho W_{j}^{*}x_{n}\rangle \\ & = & \sum_{n}\langle x_{n},O_{i}VQ^{\left(\gamma \right)}\rho Q^{\left(\gamma \right)*}V^{*}O_{j}^{*}x_{n}\rangle \\ & = & \lambda \langle i,\alpha \gamma \rangle \langle \alpha \gamma ,j\rangle +\left(1-\lambda \right)\langle i,-\beta \theta +\alpha \gamma \rangle \langle -\beta \theta +\alpha \gamma ,j\rangle \\ & = & \langle i,\lambda |\alpha \gamma \rangle \langle \alpha \gamma |+\left(1-\lambda \right)|-\beta \theta +\alpha \gamma \rangle \langle -\beta \theta +\alpha \gamma |j\rangle \end{array}$$

*Then*

$$\begin{array}{ccc} W & = & \lambda |\alpha \gamma \rangle \langle \alpha \gamma |+\left(1-\lambda \right)|-\beta \theta +\alpha \gamma \rangle \langle -\beta \theta +\alpha \gamma |\\ & = & \exp\left(\alpha \gamma a^{*}-\bar{\alpha \gamma }a\right)\left(\lambda |0\rangle \langle 0|+\left(1-\lambda \right)|-\beta \theta \rangle \langle -\beta \theta |\right)\exp\left(-\alpha \gamma a^{*}+\bar{\alpha \gamma }a\right) \end{array}$$

Based on the above theorems, one can obtain the following theorem:

**Theorem** **10.** For the noisy optical channel $\Lambda^{*}$ and the input state $\rho =\lambda |0\rangle \langle 0|+\left(1-\lambda \right)|\theta \rangle \langle \theta |$, we have

*1.*  $0\leq I\left(\rho ;\Lambda^{*}\right)\leq \min\left\{\;S\left(\rho \right),S\left(\Lambda^{*}\rho \right)\right\}$ (Ohya mutual entropy),
*2.*  $-S\left(\rho \right)\leq I_{C}\left(\rho ;\Lambda^{*}\right)\leq S\left(\rho \right)$ (coherent entropy),
*3.*  $0\leq I_{LN}\left(\rho ;\Lambda^{*}\right)\leq 2S\left(\rho \right)$ (Lindblad-Nielsen entropy).

## 7. Compound States

Based on [29], we briefly review some results concerning the entangled compound states.

When a signal is transmitted through a channel $\Lambda^{*}$ from the initial state $\rho \in S(H_{1})$ to the final state $\Lambda^{*}\rho \in S\left(H_{2}\right)$, we will consider here the methods of constructing some compound states Φ satisfying marginal conditions

$$tr_{H_{2}}\Phi =\rho \;\mathrm{and}\;tr_{H_{1}}\Phi =\Lambda^{*}\rho$$

For the initial state ρ, let $\sum_{n}\lambda_{n}E_{n}$ be the Schatten - von Neumann decomposition of ρ, which is not unique if the eigenvalues are degenerate. The following separable compound states with respect to the input state ρ and the quantum channel $\Lambda^{*}$ satisfies the marginal conditions.

$$\begin{array}{ccc} \sigma_{E} & = & \sum_{n}\lambda_{n}E_{n}⊗\Lambda^{*}E_{n}\;\left(\mathrm{Ohya}\;\mathrm{compound}\;\mathrm{state}\right),\\ \sigma_{0} & = & {\rho ⊗\Lambda }^{*}\rho \;\left(\mathrm{trivial}\;\mathrm{compound}\;\mathrm{state}\right). \end{array}$$

Let $V_{k}$$\left(\forall k\right)$ be a linear mapping from $H_{1}$ to $H_{2}$. For the CP channel $\Lambda^{*}$ represented by the Stinespring-Sudarshan-Kraus form as

$$\Lambda^{*}\left(\rho \right)=\sum_{k}V_{k}\rho V_{k}^{*}\;\mathrm{with}\;\sum_{k}V_{k}^{*}V_{k}=I,$$

$\sigma_{0}$ and $\sigma_{E}$ are obtained by using the Jamiołkowski isomorphism channel [25]

$$\begin{array}{ccc} \sigma_{0} & = & \sum_{k}\left(I⊗V_{k}\right)\left(\omega \right)\left(I⊗V_{k}^{*}\right),\\ \sigma_{E} & = & \sum_{k}\left(I⊗V_{k}\right)\left(\omega_{E}\right)\left(I⊗V_{k}^{*}\right), \end{array}$$

where ω and $\omega_{E}$ are the separable compound states given by

$$\begin{array}{ccc} \omega & = & \rho ⊗\rho ,\\ \omega_{E} & = & \sum_{n}\lambda_{n}E_{n}⊗E_{n}. \end{array}$$

The point I wish to emphasize is that what kind of compound state is most suitable for discussing the efficiency of information transmission for the quantum communication processes including the entangled physical phenomenon. A great deal of effort has been made on this problem. What seems to be lacking, however, is to investigate this problem as a whole. Therefore I discuss this problem as a whole repeating these theorems in this paper in addition to new theorems.

For the quantum channel $\Lambda^{*}$ and the Schatten decomposition of $\rho =\sum_{k}\lambda_{k}E_{k}$, let $\Psi_{E}$ be a compound state defined by

$$\begin{array}{ccc} \Psi_{E} & = & \sum_{n}\left(I⊗V_{n}\right)\left[\sum_{k}\sqrt{\lambda_{k}}\left(|x_{k}\rangle ⊗|x_{k}\rangle \right)\right]\\ & & \cdot \left[\sum_{k^{\prime }}\sqrt{\lambda_{k^{\prime }}}\left(\langle x_{k^{\prime }}|⊗\langle x_{k^{\prime }}|\right)\right]\left(I⊗V_{n}^{*}\right) \end{array}$$

satisfying

$$\sum_{n}\left(I⊗V_{n}^{*}\right)\left(I⊗V_{n}\right)=I⊗I.$$

Base on [29], one has the following theorem.

**Theorem** **11.** Let $\Psi_{E}$ be an entangled compound state with respect to the input state ρ, the CP channel $\Lambda^{*}$ and the Schatten - von Neumann decomposition $\rho =\sum_{n}\lambda_{n}E_{n}$ of ρ defined by

$$\begin{array}{ccc} \Psi_{E} & = & \sum_{k}\left(I⊗V_{k}\right)\left[\sum_{n}\sqrt{\lambda_{n}}|x_{n}\rangle ⊗|x_{n}\rangle \right]\\ & & \cdot \left[\sum_{n^{\prime }}\sqrt{\lambda_{n^{\prime }}}\langle x_{n^{\prime }}|⊗\langle x_{n^{\prime }}|\right]\left(I⊗V_{k}^{*}\right) \end{array}$$

*under the condition*

$$\sum_{k}\left(I⊗V_{k}\right)\left(I⊗V_{k}^{*}\right)=I⊗I\;\mathrm{and}\;\;\Lambda^{*}\left(\rho \right)=\sum_{k}V_{k}\rho V_{k}^{*}.$$

*Then $\Psi_{E}$ holds two marginal condition*

$$tr_{H_{2}}\Psi_{E}=\rho \;and\;\;tr_{H_{1}}\Psi_{E}=\Lambda^{*}\left(\rho \right)$$

*and the upper bound of the relative entropy between $\Psi_{E}$ and $\sigma_{0}$ is given by*

$$S\left(\Psi_{E},\sigma_{0}\right)\leq 2S\left(\rho \right).$$

**Proof.**  $$\begin{array}{cc} & \langle x,tr_{H_{2}}\Psi_{E}x^{\prime }\rangle \\ = & \sum_{m}\langle x⊗y_{m},\Psi_{E}x^{\prime }⊗y_{m}\rangle \\ = & \sum_{m}\sum_{n}\sum_{k}\sum_{k^{\prime }}\sqrt{\lambda_{k}}\sqrt{\lambda_{k^{\prime }}}\langle x,|x_{k}\rangle \langle x_{k^{\prime }}|x^{\prime }\rangle \langle y_{m},V_{n}|x_{k}\rangle \langle x_{k^{\prime }}|V_{n}^{*}y_{m}\rangle \\ = & \sum_{k}\sum_{k^{\prime }}\sqrt{\lambda_{k}}\sqrt{\lambda_{k^{\prime }}}\langle x,|x_{k}\rangle \langle x_{k^{\prime }}|x^{\prime }\rangle \langle x_{k^{\prime }},x_{k}\rangle \\ = & \sum_{k}\lambda_{k}\langle x,|x_{k}\rangle \langle x_{k}|x^{\prime }\rangle \\ = & \langle x,\left(\sum_{k}\lambda_{k}|x_{k}\rangle \langle x_{k}|\right)x^{\prime }\rangle \\ = & \langle x,\rho x^{\prime }\rangle \end{array}$$

for any $x,x^{\prime }$ in $H_{1}$. Then one has

$$tr_{H_{2}}\Psi_{E}=\rho .$$

$$\begin{array}{cc} & \langle y,tr_{H_{1}}\Psi_{E}y^{\prime }\rangle \\ = & \sum_{i}\langle x_{i}⊗y,\Psi_{E}x_{i}⊗y^{\prime }\rangle \\ = & \sum_{i}\sum_{n}\sum_{k}\sum_{k^{\prime }}\sqrt{\lambda_{k}}\sqrt{\lambda_{k^{\prime }}}\langle x_{i},x_{k}\rangle \langle x_{k^{\prime }},x_{i}\rangle \langle y,V_{n}|x_{k}\rangle \langle x_{k^{\prime }}|V_{n}^{*}y^{\prime }\rangle \\ = & \sum_{n}\sum_{k}\lambda_{k}\langle y,V_{n}|x_{k}\rangle \langle x_{k}|V_{n}^{*}y^{\prime }\rangle \\ = & \langle y,\sum_{n}V_{n}\left(\sum_{k}\lambda_{k}|x_{k}\rangle \langle x_{k}|\right)V_{n}^{*}y^{\prime }\rangle \\ = & \langle y,\Lambda^{*}\rho y^{\prime }\rangle \end{array}$$

for any $y,y^{\prime }$ in $H_{2}$. Then we have

$$tr_{H_{1}}\Psi_{E}=\Lambda^{*}\rho .$$

After simple calculation, we have

$$\begin{array}{cc} & S\left(\Psi_{E},\rho ⊗\Lambda^{*}\rho \right)\\ \leq & S\left(\left[\sum_{k}\sqrt{\lambda_{k}}\left(|x_{k}\rangle ⊗|x_{k}\rangle \right)\right]\left[\sum_{k^{\prime }}\sqrt{\lambda_{k^{\prime }}}\left(\langle x_{k^{\prime }}|⊗\langle x_{k^{\prime }}|\right)\right],\right.\\ & \left.,\left[\sum_{k}\lambda_{k}E_{k}⊗\rho \right]\right.\\ = & -\sum_{n,m}\sum_{k}\sqrt{\lambda_{k}}\langle x_{n}⊗x_{m},x_{k}⊗x_{k}\rangle \\ & \sum_{k^{\prime }}\sqrt{\lambda_{k^{\prime }}}\langle x_{k^{\prime }}⊗x_{k^{\prime }},\log\left[\rho ⊗\rho \right]x_{n}⊗x_{m}\rangle \\ = & -2\sum_{k}\lambda_{k}\langle x_{k},\left[\log\rho \right]x_{k}\rangle \\ = & 2S\left(\rho \right) \end{array}$$

□

Then one has the following results [29].

**Corollary** **1.** Let $\Phi_{E}$ be a pure entangled compound state with respect to the input state ρ, the CP channel $\Lambda^{*}$ and the Schatten - von Neumann decomposition $\rho =\sum_{n}\lambda_{n}E_{n}$ of ρ defined by

$$\begin{array}{ccc} \Phi_{E} & = & \left(I⊗V\right)\left[\sum_{n}\sqrt{\lambda_{n}}|x_{n}\rangle ⊗|x_{n}\rangle \right]\\ & & \cdot \left[\sum_{n^{\prime }}\sqrt{\lambda_{n^{\prime }}}\langle x_{n^{\prime }}|⊗\langle x_{n^{\prime }}|\right]\left(I⊗V^{*}\right) \end{array}$$

*under the condition*

$$\left(I⊗V\right)\left(I⊗V^{*}\right)=I⊗I\;\mathrm{and}\;\;\Lambda^{*}\left(\rho \right)=V\rho V^{*}.$$

*Then $\Psi_{E}$ holds two marginal condition*

$$tr_{H_{2}}\Phi_{E}=\rho \;\mathrm{and}\;\;tr_{H_{1}}\Phi_{E}=\Lambda^{*}\left(\rho \right)$$

*and the upper bound of the relative entropy between $\Phi_{E}$ and $\sigma_{0}$ is given by*

$$S\left(\Phi_{E},\sigma_{0}\right)\leq 2S\left(\rho \right).$$

**Corollary** **2.** Let $\Psi_{E}$ be a mixed entangled compound state with respect to the input state ρ, the CP channel $\Lambda^{*}$ and the Schatten - von Neumann decomposition $\rho =\sum_{n}\lambda_{n}E_{n}$ of ρ defined by

$$\begin{array}{ccc} \Psi_{E}^{\prime } & = & \sum_{k}\left(I⊗V_{k}\right)\left[\sum_{n}\sqrt{\lambda_{n}}|x_{n}\rangle ⊗|x_{n}\rangle \right]\\ & & \cdot \left[\sum_{n^{\prime }}\sqrt{\lambda_{n^{\prime }}}\langle x_{n^{\prime }}|⊗\langle x_{n^{\prime }}|\right]\left(I⊗V_{k}^{*}\right) \end{array}$$

*under the condition $V_{k}=\sum_{j}\mu_{k}|y_{j}^{\left(k\right)}\rangle \langle x_{j}|$*

$$\sum_{k}\left(I⊗V_{k}\right)\left(I⊗V_{k}^{*}\right)=I⊗I\;\mathrm{and}\;\;\Lambda^{*}\left(\rho \right)=\sum_{k}V_{k}\rho V_{k}^{*}.$$

*$\Psi_{E}^{\prime }$ holds two marginal condition*

$$tr_{H_{2}}\Psi_{E}^{\prime }=\rho \;\mathrm{and}\;\;tr_{H_{1}}\Psi_{E}^{\prime }=\Lambda^{*}\left(\rho \right).$$

*If $y_{i}^{\left(k\right)}⊥$$y_{j}^{\left(k^{\prime }\right)}$$\left(\mathrm{for}\;\mathrm{any}\;i,j,\;k\neq k^{\prime }\right)$ holds, then the upper bound of the relative entropy between $\Psi_{E}^{\prime }$ and $\sigma_{0}$ is given by*

$$S\left(\Psi_{E}^{\prime },\sigma_{0}\right)\leq 2S\left(\rho \right).$$

The following results are obtained for the compound state given by the affine combination of the separable and entangled compound states. [29].

**Theorem** **12.** *For any $\mu \in \left[0,1\right],$ let $\Psi_{E,\mu }$ be a compound state defined by*

$$\Psi_{E,\mu }=\mu \sigma_{E}+\left(1-\mu \right)\Psi_{E}.$$

*$\Psi_{E,\mu }$ satisfies two marginal conditions as follows:*

$$tr_{H_{2}}\Psi_{E,\mu }=\rho \;\mathrm{and}\;\;tr_{H_{1}}\Psi_{E,\mu }=\Lambda^{*}\left(\rho \right).$$

*One can obtain the upper bound of the relative entropy between $\Psi_{E,\mu }$ and $\sigma_{0}$*

$$S\left(\Psi_{E,\mu },\sigma_{0}\right)\leq \left(2-\mu \right)S\left(\rho \right).$$

According to [29], the relation between the separable and the entangled compound states is satisfied.

**Theorem** **13.** There exists a CP channel $\Xi^{*}$ depending on the Schatten - von Neumann decomposition of the input state ρ from the entangled compound state $\Delta_{E}$

$$\Delta_{E}=\left[\sum_{n}\sqrt{\lambda_{n}}|x_{n}\rangle ⊗|x_{n}\rangle \right]\left[\sum_{n^{\prime }}\sqrt{\lambda_{n^{\prime }}}\langle x_{n^{\prime }}|⊗\langle x_{n^{\prime }}|\right]$$

*to the separable compound state $\omega_{E}=\sum_{n}\lambda_{n}E_{n}⊗E_{n}$ as follows:*

$$\Xi^{*}\left(•\right)=\sum_{n,n^{\prime }}W_{n,n^{\prime }}\left(•\right)W_{n,n^{\prime }}^{*},$$

*where $W_{n,n^{\prime }}$ is given by*

$$W_{n,n^{\prime }}=|x_{n}\rangle \langle x_{n}|⊗|x_{n^{\prime }}\rangle \langle x_{n^{\prime }}|$$

*satisfying*

$$\sum_{n,n^{\prime }}W_{n,n^{\prime }}^{*}W_{n,n^{\prime }}=I⊗I.$$

**Theorem** **14.** There exists a CP channel $\Xi^{*}$ depending on the Schatten-von Neumann decomposition of the input state ρ from the separable compound state $\omega_{E}=\sum_{n}\lambda_{n}E_{n}⊗E_{n}$ to the entangled compound state $\Delta_{E}$

$$\Delta_{E}=\left[\sum_{n}\sqrt{\lambda_{n}}|x_{n}\rangle ⊗|x_{n}\rangle \right]\left[\sum_{n^{\prime }}\sqrt{\lambda_{n^{\prime }}}\langle x_{n^{\prime }}|⊗\langle x_{n^{\prime }}|\right]$$

*as follows:*

$${\tilde{\Xi }}^{*}\left(•\right)=\sum_{n,n^{\prime }}w_{n,n^{\prime }}\left(•\right)w_{n,n^{\prime }}^{*},$$

*where $w_{n,n^{\prime }}$ is given by*

$$W_{n,n^{\prime }}=\left(\sum_{k}\sqrt{\lambda_{k}}|x_{k}\rangle ⊗|x_{k}\rangle \right)\langle x_{n}|⊗\langle x_{n^{\prime }}|$$

*with the condition*

$$\sum_{n,n^{\prime }}w_{n,n^{\prime }}^{*}w_{n,n^{\prime }}=I⊗I.$$

Based on [29], one obtains the following theorems for the attenuation channel $\Lambda_{0}^{*}$.

**Theorem** **15.** For the attenuation channel $\Lambda_{0}^{*}$ and the input state

$$\rho =\lambda |0\rangle \langle 0|+\left(1-\lambda \right)|\theta \rangle \langle \theta |,$$

*if $\lambda =\frac{1}{2}$ and $\beta =\sqrt{\frac{2}{3}}$, then there exists a compound state *Φ* satisfying*

$$I\left(\rho ;\Lambda_{0}^{*}\right)=S\left(\Phi ,\rho ⊗\Lambda_{0}^{*}\rho \right)=S\left(\rho \right)+S\left(\Lambda_{0}^{*}\rho \right)+trW\log W,$$

*where W is a matrix $W={\left(W_{ij}\right)}_{i,\;j}$ with*

$$W_{ij}\equiv trA_{i}^{*}\rho A_{j}$$

*for a state ρ concerning a Stinespring-Sudarshan-Kraus form*

$$\Lambda_{0}^{*}\left(\cdot \right)\equiv \sum_{j}A_{j}^{*}\cdot A_{j},$$

of a channel $\Lambda_{0}^{*}$.

**Theorem** **16.** For the attenuation channel $\Lambda_{0}^{*}$ and the input state

$$\rho =\lambda |0\rangle \langle 0|+\left(1-\lambda \right)|\theta \rangle \langle \theta |,$$

*if $\lambda =\frac{1}{2}$ and α=1, then there exists a compound state *Φ* satisfying*

$$S\left(\Phi ,\rho ⊗\Lambda_{0}^{*}\rho \right)=S\left(\rho \right).$$

Here, we introduce the construction of the hybrid entangled compound state $\Phi_{E}^{\left(\Delta \right)}$ as follow: For an initial state ρ, the Schatten decomposition of ρ is given by

$$\rho =\sum_{n\in Q}\lambda_{n}E_{n},$$

where *Q* is the total index set with respect to a decomposition of the state. One can create a compound state $\Phi_{E}^{\left(\Delta \right)}$ with respect to a subset Δ of *Q* as

$$\begin{array}{ccc} \Phi_{E}^{\left(\Delta \right)} & = & \left(\sum_{n_{i}\in \Delta }\sqrt{\lambda_{n_{i}}}|x_{n_{i}}\rangle ⊗|x_{n_{i}}\rangle \right)\left(\sum_{n_{j}\in \Delta }\sqrt{\lambda_{n_{j}}}\langle x_{n_{j}}|⊗\langle x_{n_{j}}|\right)\\ & & +\sum_{n_{k}\in Q\backslash \Delta }\lambda_{n_{k}}E_{n_{k}}⊗E_{n_{k}} \end{array}$$

If the cardinality $\#\left(\Delta \right)$ of subset Δ of *Q* holds $\#\left(\Delta \right)\leq 1$, then $\Phi_{E}^{\left(1\right)}$ is called a separable compound state denoted by

$$\Phi_{E}^{\left(1\right)}=\sum_{n_{k}\in Q}\lambda_{n_{k}}E_{n_{k}}⊗E_{n_{k}}.$$

If Δ=Q is held, then $\Phi_{E}^{\left(Q\right)}$ is called a full entangled compound state denoted by

$$\Phi_{E}^{\left(Q\right)}=\left(\sum_{n_{i}\in Q}\sqrt{\lambda_{n_{i}}}|x_{n_{i}}\rangle ⊗|x_{n_{i}}\rangle \right)\left(\sum_{n_{j}\in Q}\sqrt{\lambda_{n_{j}}}\langle x_{n_{j}}|⊗\langle x_{n_{j}}|\right).$$

If $2\leq \#\left(\Delta \right)<\#\left(Q\right)$ is held, then $\Phi_{E}^{\left(\Delta \right)}$ is called a hybrid compound state concerned with an index set Δ denoted by

$$\begin{array}{ccc} \Phi_{E}^{\left(\Delta \right)} & = & \left(\sum_{n_{i}\in \Delta }\sqrt{\lambda_{n_{i}}}|x_{n_{i}}\rangle ⊗|x_{n_{i}}\rangle \right)\left(\sum_{n_{j}\in \Delta }\sqrt{\lambda_{n_{j}}}\langle x_{n_{j}}|⊗\langle x_{n_{j}}|\right)\\ & & +\sum_{n_{k}\in Q\backslash \Delta }\lambda_{n_{k}}E_{n_{k}}⊗E_{n_{k}} \end{array}$$

Let us consider the completely positive channel $\Lambda_{t}^{*}$ given by $\Lambda_{t}^{*}\left(\rho \right)=V_{t}\rho V_{t}^{*}$ for any $\rho \in S\left(H_{1}\right),$t≥0 with $V_{t}^{*}V_{t}=I$ and $\lim_{t\to 0}V_{t}=\lim_{t\to 0}V_{t}^{*}=I.$

By using the Jamiolkowski isomorphic channel one can define the following compound states:

(1) The separable compound state $\Psi_{E,\Lambda_{t}^{*}}^{\left(1\right)}$ with respect to the Schatten decomposition $\sum_{n\in Q}\lambda_{n}E_{n}$ of the initial state ρ and the completely positive channel $\Lambda_{t}^{*}$ is defined by

$$\begin{array}{ccc} \Psi_{E,\Lambda_{t}^{*}}^{\left(1\right)} & = & \left(id⊗V_{t}\right)\Phi_{E}^{\left(1\right)}\left(id⊗V_{t}^{*}\right)\\ & = & \sum_{n_{k}\in Q}\lambda_{n_{k}}E_{n_{k}}⊗\Lambda_{t}^{*}\left(E_{n_{k}}\right). \end{array}$$

(2) The full entangled compound state $\Psi_{E,\Lambda_{t}^{*}}^{\left(Q\right)}$ with respect to the Schatten decomposition of the initial state ρ and the completely positive channel $\Lambda_{t}^{*}$ is defined by

$$\begin{array}{ccc} \Psi_{E,\Lambda_{t}^{*}}^{\left(Q\right)} & = & \left(id⊗V_{t}\right)\Phi_{E}^{\left(Q\right)}\left(id⊗V_{t}^{*}\right)\\ & = & \left(\sum_{n_{i}\in Q}\sqrt{\lambda_{n_{i}}}|x_{n_{i}}\rangle ⊗V_{t}|x_{n_{i}}\rangle \right)\left(\sum_{n_{j}\in Q}\sqrt{\lambda_{n_{j}}}\langle x_{n_{j}}|⊗\langle x_{n_{j}}|V_{t}^{*}\right). \end{array}$$

(3) The hybrid compound state $\Psi_{E,\Lambda_{t}^{*}}^{\left(\Delta \right)}$ concerned with an index set Δ with respect to the Schatten decomposition of the initial state ρ and the completely positive channel $\Lambda_{t}^{*}$ is defined by

$$\begin{array}{ccc} \Psi_{E,\Lambda_{t}^{*}}^{\left(\Delta \right)} & = & \left(id⊗V_{t}\right)\Phi_{E}^{\left(\Delta \right)}\left(id⊗V_{t}^{*}\right)\\ & = & \left(\sum_{n_{i}\in \Delta }\sqrt{\lambda_{n_{i}}}|x_{n_{i}}\rangle ⊗V_{t}|x_{n_{i}}\rangle \right)\left(\sum_{n_{j}\in \Delta }\sqrt{\lambda_{n_{j}}}\langle x_{n_{j}}|⊗\langle x_{n_{j}}|V_{t}^{*}\right)\\ & & +\sum_{n_{k}\in Q\backslash \Delta }\lambda_{n_{k}}E_{n_{k}}⊗\Lambda_{t}^{*}\left(E_{n_{k}}\right) \end{array}$$

Please note that one can define the hybrid compound state $\Psi_{E,\Lambda_{t}^{*}}^{\left(\Delta \right)}$ by using the compound lifting $E_{E\left(\Delta \right),\Lambda_{t}^{*}}^{*}$ such that

$$\begin{array}{ccc} E_{E\left(\Delta \right),\Lambda_{t}^{*}}^{*}\left(\rho \right) & = & \left(\sum_{n_{i}\in \Delta }\sqrt{\lambda_{n_{i}}}|x_{n_{i}}\rangle ⊗V_{t}|x_{n_{i}}\rangle \right)\left(\sum_{n_{j}\in \Delta }\sqrt{\lambda_{n_{j}}}\langle x_{n_{j}}|⊗\langle x_{n_{j}}|V_{t}^{*}\right)\\ & & +\sum_{n_{k}\in Q\backslash \Delta }\lambda_{n_{k}}E_{n_{k}}⊗\Lambda_{t}^{*}\left(E_{n_{k}}\right). \end{array}$$

We define the mutual entropy type measure as follows: For a Schatten decomposition $\sum_{n\in Q}\lambda_{n}E_{n}$ of the initial state ρ, let $\Psi_{E,\Lambda_{t}^{*}}^{\left(\Delta \right)}$ be an entangled compound state with respect to a subset Δ⊂Q$\left(\#\left(\Delta \right)\geq 2\right)$ and the CP channel $\Lambda_{t}^{*}\left(\rho \right)=V_{t}\rho V_{t}^{*}$ for any $\rho \in S\left(H_{1}\right),$t≥0 with $V_{t}^{*}V_{t}=I$ and $\lim_{t\to 0}V_{t}=\lim_{t\to 0}V_{t}^{*}=I$. The mutual entropy type measure $I^{\left(\Delta \right)}\left(\rho ;\Lambda_{t}^{*}\right)$ with respect to a subset Δ⊂Q$\left(\#\left(\Delta \right)\geq 2\right)$ and the CP channel $\Lambda_{t}^{*}$ is defined by taking the supremum of the relative entropy between $\Psi_{E,\Lambda_{t}^{*}}^{\left(\Delta \right)}$ and $\rho ⊗\Lambda_{t}^{*}\rho$ for all Schatten decomposition $E=\left\{E_{n}\right\}$ of the initial state ρ

$$I^{\left(\Delta \right)}\left(\rho ;\Lambda_{t}^{*}\right)=\sup\left\{S\left(\Psi_{E,\Lambda_{t}^{*}}^{\left(\Delta \right)},\rho ⊗\Lambda_{t}^{*}\rho \right);\;E=\left\{E_{n}\right\}\right\}.$$

**Theorem** **17.** For a Schatten decomposition $\sum_{n\in Q}\lambda_{n}E_{n}$ of the initial state ρ, let $\Psi_{E,\Lambda_{t}^{*}}^{\left(\Delta \right)}$ be an entangled compound state with respect to a subset Δ⊂Q$\left(\#\left(\Delta \right)\geq 2\right)$ and the CP channel $\Lambda_{t}^{*}\left(\rho \right)=V_{t}\rho V_{t}^{*}$ for any $\rho \in S\left(H_{1}\right),$t≥0 with $V_{t}^{*}V_{t}=I$ and $\lim_{t\to 0}V_{t}=\lim_{t\to 0}V_{t}^{*}=I$ It holds two marginal conditions

$$tr_{H_{2}}\Psi_{E,\Lambda_{t}^{*}}^{\left(\Delta \right)}=\rho \;\mathrm{and}\;\;tr_{H_{1}}\Psi_{E,\Lambda_{t}^{*}}^{\left(\Delta \right)}=\Lambda_{t}^{*}\left(\rho \right)$$

and the relative entropy between $\Psi_{E,\Lambda_{t}^{*}}^{\left(\Delta \right)}$ and $\sigma_{0}=\rho ⊗\Lambda_{t}^{*}\rho$ satisfies the following inequality:

$$I^{\left(\Delta \right)}\left(\rho ;\Lambda_{t}^{*}\right)\geq S\left(\Psi_{E,\Lambda_{t}^{*}}^{\left(\Delta \right)},\rho ⊗\Lambda_{t}^{*}\left(\rho \right)\right)>S\left(\Lambda_{t}^{*}\rho \right).$$

**Proof.**  Since

$$\begin{array}{cc} & \Psi_{E,\Lambda_{t}^{*}}^{\left(\Delta \right)}\\ = & \left(I⊗V_{t}\right)\left[\sum_{n_{i}\in \Delta }\sqrt{\lambda_{n_{i}}}|x_{n_{i}}\rangle ⊗|x_{n_{i}}\rangle \right]\\ & \cdot \left[\sum_{n_{j}\in \Delta }\sqrt{\lambda_{n_{j}}}\langle x_{n_{j}}|⊗\langle x_{n_{j}}|\right]\left(I⊗V_{t}^{*}\right)\\ & +\left(I⊗V_{t}\right)\left[\sum_{n_{k}\in Q\backslash \Delta }\lambda_{n_{k}}E_{n_{k}}⊗E_{n_{k}}\right]\left(I⊗V_{t}^{*}\right)\\ = & {∥\phi_{\Delta }∥}^{2}\frac{|\phi_{\Delta }\rangle }{∥\phi_{\Delta }∥}\frac{\langle \phi_{\Delta }|}{∥\phi_{\Delta }∥}+\sum_{n_{k}\in Q\backslash \Delta }\lambda_{n_{k}}E_{n_{k}}⊗V_{t}E_{n_{k}}V_{t}^{*} \end{array}$$

is held, then one has

$$\begin{array}{cc} & tr\Psi_{E,\Lambda_{t}^{*}}^{\left(\Delta \right)}\log\Psi_{E,\Lambda_{t}^{*}}^{\left(\Delta \right)}\\ = & {∥\phi_{\Delta }∥}^{2}\log{∥\phi_{\Delta }∥}^{2}+\sum_{n_{k}\in Q\backslash \Delta }\lambda_{n_{k}}\log\lambda_{n_{k}}\\ = & \left(\sum_{n_{i}\in \Delta }\lambda_{n_{i}}\right)\log\left(\sum_{n_{i}\in \Delta }\lambda_{n_{i}}\right)+\sum_{n_{k}\in Q\backslash \Delta }\lambda_{n_{k}}\log\lambda_{n_{k}} \end{array}$$

under the condition

$$\left(I⊗V_{t}\right)\left(I⊗V_{t}^{*}\right)=I⊗I\;\mathrm{and}\;\;\Lambda_{t}^{*}\left(\rho \right)=V_{t}\rho V_{t}^{*}.$$

Then $\Psi_{E,\Lambda_{t}^{*}}^{\left(\Delta \right)}$ holds two marginal conditions

$$tr_{H_{2}}\Psi_{E,\Lambda_{t}^{*}}^{\left(\Delta \right)}=\rho \;\mathrm{and}\;\;tr_{H_{1}}\Psi_{E,\Lambda_{t}^{*}}^{\left(\Delta \right)}=\Lambda_{t}^{*}\left(\rho \right)$$

and the relative entropy between $\Psi_{E,\Lambda_{t}^{*}}^{\left(\Delta \right)}$ and $\sigma_{0}=\rho ⊗\Lambda_{t}^{*}\rho$ is obtained by

$$\begin{array}{cc} & S\left(\Psi_{E,\Lambda_{t}^{*}}^{\left(\Delta \right)},\rho ⊗\Lambda_{t}^{*}\rho \right)\\ = & S\left(\left(\sum_{n_{i}\in \Delta }\sqrt{\lambda_{n_{i}}}|x_{n_{i}}\rangle ⊗V_{t}|x_{n_{i}}\rangle \right)\left(\sum_{n_{j}\in \Delta }\sqrt{\lambda_{n_{j}}}\langle x_{n_{j}}|⊗\langle x_{n_{j}}|V_{t}^{*}\right)\right.\\ & \left.+\left(\sum_{n_{k}\in Q\backslash \Delta }\lambda_{n_{k}}E_{n_{k}}⊗V_{t}\rho V_{t}^{*}\right),\rho ⊗\Lambda_{t}^{*}\rho \right)\\ = & tr\Psi_{E,\Lambda_{t}^{*}}^{\left(\Delta \right)}\log\Psi_{E,\Lambda_{t}^{*}}^{\left(\Delta \right)}-tr\Psi_{E,\Lambda_{t}^{*}}^{\left(\Delta \right)}\log\rho ⊗\Lambda_{t}^{*}\rho \\ = & S\left(\rho \right)+S\left(\Lambda_{t}^{*}\rho \right)+{∥\phi_{\Delta }∥}^{2}\log{∥\phi_{\Delta }∥}^{2}+\sum_{n_{k}\in Q\backslash \Delta }\lambda_{n_{k}}\log\lambda_{n_{k}}\\ = & -\sum_{n_{k}\in \Delta }\lambda_{n_{k}}\log\lambda_{n_{k}}+S\left(\Lambda_{t}^{*}\rho \right)+\left(\sum_{n_{k}\in \Delta }\lambda_{n_{k}}\right)\log\left(\sum_{n_{k}\in \Delta }\lambda_{n_{k}}\right)\\ = & -\sum_{n_{k}\in \Delta }\lambda_{n_{k}}\log\frac{\lambda_{n_{k}}}{\left(\sum_{n_{k}\in \Delta }\lambda_{n_{k}}\right)}+S\left(\Lambda_{t}^{*}\rho \right)>S\left(\Lambda_{t}^{*}\rho \right) \end{array}$$

Therefore, we get the following inequality:

$$I^{\left(\Delta \right)}\left(\rho ;\Lambda_{t}^{*}\right)\geq S\left(\Psi_{E,\Lambda_{t}^{*}}^{\left(\Delta \right)},\rho ⊗\Lambda_{t}^{*}\left(\rho \right)\right)>S\left(\Lambda_{t}^{*}\rho \right).$$

□

It shows that the mutual entropy at time *t* defined by using the entangled compound state greater than the von Neumann entropy $S\left(\Lambda_{t}^{*}\rho \right)$ of the final state $\Lambda_{t}^{*}\rho$. When t→0 is held, one has the following inequality

$$I^{\left(\Delta \right)}\left(\rho ;id\right)\geq \lim_{t\to 0}S\left(\Psi_{E,\Lambda_{t}^{*}}^{\left(\Delta \right)},\rho ⊗\Lambda_{t}^{*}\rho \right)>S\left(\rho \right)$$

It means that the mutual entropy type measure defined by using the entangled compound state at initial time t=0 greater than the von Neumann entropy $S\left(\rho \right)$ of the initial state ρ.

Let $\Lambda^{*}$ be a completely positive channels $\Lambda^{*}$ given by

$$\Lambda^{*}\left(\rho \right)=\sum_{n}V_{n}\left(\rho \right)V_{n}^{*}$$

satisfying

$$\sum_{n}V_{n}^{*}V_{n}=I\;$$

(1) The separable compound state $\Psi_{E,\Lambda_{t}^{*}}^{\left(1\right)}$ with respect to the Schatten decomposition $\sum_{n\in Q}\lambda_{n}E_{n}$ of the initial state ρ and the completely positive channel $\Lambda^{*}$ is defined by

$$\begin{array}{ccc} \Psi_{E,\Lambda^{*}}^{\left(1\right)} & = & \sum_{n}\left(id⊗V_{n}\right)\Phi_{E}^{\left(1\right)}\left(id⊗V_{n}^{*}\right)\\ & = & \sum_{n_{k}\in Q}\lambda_{n_{k}}E_{n_{k}}⊗\Lambda^{*}\left(E_{n_{k}}\right). \end{array}$$

(2) The full entangled compound state $\Psi_{E,\Lambda_{t}^{*}}^{\left(Q\right)}$ with respect to the Schatten decomposition of the initial state ρ and the completely positive channel $\Lambda^{*}$ is defined by

$$\begin{array}{cc} & \Psi_{E,\Lambda^{*}}^{\left(Q\right)}\\ = & \sum_{n}\left(id⊗V_{n}\right)\Phi_{E}^{\left(Q\right)}\left(id⊗V_{n}^{*}\right)\\ = & \sum_{n}\left(\sum_{n_{i}\in Q}\sqrt{\lambda_{n_{i}}}|x_{n_{i}}\rangle ⊗V_{n}|x_{n_{i}}\rangle \right)\left(\sum_{n_{j}\in Q}\sqrt{\lambda_{n_{j}}}\langle x_{n_{j}}|⊗\langle x_{n_{j}}|V_{n}^{*}\right). \end{array}$$

(3) The hybrid compound state $\Psi_{E,\Lambda^{*}}^{\left(\Delta \right)}$ with respect to a subset Δ⊂Q$\left(\#\left(\Delta \right)\geq 2\right)$, the Schatten decomposition of the initial state ρ and the completely positive channel $\Lambda^{*}$ is defined by

$$\begin{array}{cc} & \Psi_{E,\Lambda^{*}}^{\left(\Delta \right)}\\ = & \sum_{n}\left(id⊗V_{n}\right)\Phi_{E}^{\left(\Delta \right)}\left(id⊗V_{n}^{*}\right)\\ = & \sum_{n}\left(\sum_{n_{i}\in \Delta }\sqrt{\lambda_{n_{i}}}|x_{n_{i}}\rangle ⊗V_{n}|x_{n_{i}}\rangle \right)\left(\sum_{n_{j}\in \Delta }\sqrt{\lambda_{n_{j}}}\langle x_{n_{j}}|⊗\langle x_{n_{j}}|V_{n}^{*}\right)\\ & +\sum_{n_{k}\in Q\backslash \Delta }\lambda_{n_{k}}E_{n_{k}}⊗\Lambda^{*}\left(E_{n_{k}}\right) \end{array}$$

Here we define the mutual entropy type measure as follows: For a Schatten decomposition $\sum_{n\in Q}\lambda_{n}E_{n}$ of the initial state ρ, let $\Psi_{E,\Lambda^{*}}^{\left(\Delta \right)}$ be an entangled compound state with respect to a subset Δ⊂Q$\left(\#\left(\Delta \right)\geq 2\right)$ and the CP channel $\Lambda^{*}\left(\rho \right)=\sum_{n}V_{n}\left(\rho \right)V_{n}^{*}$ for any $\rho \in S\left(H_{1}\right)$ with $\sum_{n}V_{n}^{*}V_{n}=I$ and $V_{m}^{*}V_{n}=\delta_{mn}V_{n}^{*}V_{n}$. The mutual entropy type measure $I^{\left(\Delta \right)}\left(\rho ;\Lambda^{*}\right)$ with respect to a subset Δ⊂Q $\left(\#\left(\Delta \right)\geq 2\right)$ and the CP channel $\Lambda^{*}$ is defined by taking the supremum of the relative entropy between $\Psi_{E,\Lambda^{*}}^{\left(\Delta \right)}$ and $\rho ⊗\Lambda^{*}\rho$ for all Schatten decomposition $E=\left\{E_{n}\right\}$ of the initial state ρ

$$I^{\left(\Delta \right)}\left(\rho ;\Lambda^{*}\right)=\sup\left\{S\left(\Psi_{E,\Lambda^{*}}^{\left(\Delta \right)},\rho ⊗\Lambda^{*}\rho \right);\;E=\left\{E_{n}\right\}\right\}.$$

**Theorem** **18.** For a Schatten decomposition $\sum_{n\in Q}\lambda_{n}E_{n}$ of the initial state ρ, let $\Psi_{E,\Lambda^{*}}^{\left(\Delta \right)}$ be an entangled compound state with respect to a subset Δ⊂Q$\left(\#\left(\Delta \right)\geq 2\right)$ and the CP channel $\Lambda^{*}\left(\rho \right)=\sum_{n}V_{n}\left(\rho \right)V_{n}^{*}$ for any $\rho \in S\left(H_{1}\right)$ with $\sum_{n}V_{n}^{*}V_{n}=I$ and $V_{m}^{*}V_{n}=\delta_{mn}V_{n}^{*}V_{n}$. It satisfies two marginal conditions

$$tr_{H_{2}}\Psi_{E,\Lambda^{*}}^{\left(\Delta \right)}=\rho \;\mathrm{and}\;\;tr_{H_{1}}\Psi_{E,\Lambda^{*}}^{\left(\Delta \right)}=\Lambda^{*}\left(\rho \right).$$

The mutual entropy type measure $I^{\left(\Delta \right)}\left(\rho ;\Lambda^{*}\right)$ with respect to the relative entropy between $\Psi_{E,\Lambda^{*}}^{\left(\Delta \right)}$ and $\sigma_{0}=\rho ⊗\Lambda^{*}\rho$ holds the following inequality:

$$I^{\left(\Delta \right)}\left(\rho ;\Lambda^{*}\right)>I\left(\rho ;\Lambda^{*}\right),$$

where $I\left(\rho ;\Lambda^{*}\right)$ in the right-hand side is the Ohya mutual entropy.

**Proof.**  One has

$$\begin{array}{cc} & \Psi_{E,\Lambda^{*}}^{\left(\Delta \right)}\\ = & \sum_{n}\left(id⊗V_{n}\right)\left[\sum_{n_{i}\in \Delta }\sqrt{\lambda_{n_{i}}}|x_{n_{i}}\rangle ⊗|x_{n_{i}}\rangle \right]\\ & \cdot \left[\sum_{n_{j}\in \Delta }\sqrt{\lambda_{n_{j}}}\langle x_{n_{j}}|⊗\langle x_{n_{j}}|\right]\left(I⊗V_{n}^{*}\right)\\ & +\sum_{n}\left(id⊗V_{n}\right)\left[\sum_{n_{k}\in Q\backslash \Delta }\lambda_{n_{k}}E_{n_{k}}⊗E_{n_{k}}\right]\left(I⊗V_{n}^{*}\right)\\ = & \sum_{n}{∥|\phi_{n}^{\left(\Delta \right)}\rangle ∥}^{2}\frac{|\phi_{n}^{\left(\Delta \right)}\rangle }{∥|\phi_{n}^{\left(\Delta \right)}\rangle ∥}\frac{\langle \phi_{n}^{\left(\Delta \right)}|}{∥|\phi_{n}^{\left(\Delta \right)}\rangle ∥}\\ & +\sum_{n}\sum_{n_{k}\in Q\backslash \Delta }\lambda_{n_{k}}{∥V_{n}|x_{n_{k}}\rangle ∥}^{2}E_{n_{k}}⊗\frac{V_{n}|x_{n_{k}}\rangle }{∥V_{n}|x_{n_{k}}\rangle ∥}\frac{\langle x_{n_{k}}|V_{n}^{*}}{∥V_{n}|x_{n_{k}}\rangle ∥}, \end{array}$$

where

$$\begin{array}{ccc} |\phi_{n}^{\left(\Delta \right)}\rangle & = & \sum_{n_{i}\in \Delta }\sqrt{\lambda_{n_{i}}}|x_{n_{i}}\rangle ⊗V_{n}|x_{n_{i}}\rangle ,\\ {∥|\phi_{n}^{\left(\Delta \right)}\rangle ∥}^{2} & = & \sum_{n_{k}\in \Delta }\lambda_{n_{k}}{∥V_{n}|x_{n_{k}}\rangle ∥}^{2} \end{array}$$

Since

$$\begin{array}{cc} & tr\Psi_{E,\Lambda^{*}}^{\left(\Delta \right)}\log\Psi_{E,\Lambda^{*}}^{\left(\Delta \right)}\\ = & \sum_{n}\left(\sum_{n_{i}\in \Delta }\lambda_{n_{i}}{∥V_{n}|x_{n_{i}}\rangle ∥}^{2}\right)\log\left(\sum_{n_{j}\in \Delta }\lambda_{n_{j}}{∥V_{n}|x_{n_{j}}\rangle ∥}^{2}\right)\\ & +\sum_{n}\sum_{n_{k}\in Q\backslash \Delta }\lambda_{n_{k}}{∥V_{n}|x_{n_{k}}\rangle ∥}^{2}\log{∥V_{n}|x_{n_{k}}\rangle ∥}^{2}\\ & +\sum_{n_{k}\in Q\backslash \Delta }\lambda_{n_{k}}\log\lambda_{n_{k}}, \end{array}$$

under the condition

$$\begin{array}{ccc} \sum_{n}\left(I⊗V_{n}\right)\left(I⊗V_{n}^{*}\right) & = & I⊗I\\ \;\mathrm{and}\;\;\Lambda^{*}\left(\rho \right) & = & \sum_{n}V_{n}\rho V_{n}^{*}\;\mathrm{for}\;\mathrm{any}\;\rho \in S\left(H_{1}\right)\;\\ \mathrm{with}\;\sum_{n}V_{n}^{*}V_{n} & = & I\;\mathrm{and}\;V_{m}^{*}V_{n}=\delta_{mn}V_{n}^{*}V_{n} \end{array}$$

$\Psi_{E,\Lambda^{*}}^{\left(\Delta \right)}$ holds two marginal conditions

$$tr_{H_{2}}\Psi_{E,\Lambda^{*}}^{\left(\Delta \right)}=\rho \;\mathrm{and}\;\;tr_{H_{1}}\Psi_{E,\Lambda^{*}}^{\left(\Delta \right)}=\Lambda^{*}\left(\rho \right).$$

The relative entropy between $\Psi_{E,\Lambda^{*}}^{\left(\Delta \right)}$ and $\sigma_{0}=\rho ⊗\Lambda^{*}\rho$ is obtained by

$$\begin{array}{cc} & S\left(\Psi_{E,\Lambda^{*}}^{\left(\Delta \right)},\rho ⊗\Lambda^{*}\rho \right)\\ = & tr\Psi_{E,\Lambda^{*}}^{\left(\Delta \right)}\log\Psi_{E,\Lambda^{*}}^{\left(\Delta \right)}-tr\Psi_{E,\Lambda^{*}}^{\left(\Delta \right)}\log\rho ⊗\Lambda^{*}\rho \\ = & S\left(\Lambda^{*}\rho \right)-\sum_{n_{k}\in Q}\lambda_{n_{k}}S\left(\Lambda^{*}E_{n_{k}}\right)\\ & -\sum_{n}\sum_{n_{i}\in \Delta }\lambda_{n_{i}}{∥V_{n}|x_{n_{i}}\rangle ∥}^{2}\log\frac{\lambda_{n_{i}}{∥V_{n}|x_{n_{i}}\rangle ∥}^{2}}{\left(\sum_{n_{j}\in \Delta }\lambda_{n_{j}}{∥V_{n}|x_{n_{j}}\rangle ∥}^{2}\right)} \end{array}$$

Thus, we have the inequality

$$I^{\left(\Delta \right)}\left(\rho ;\Lambda^{*}\right)>I\left(\rho ;\Lambda^{*}\right).$$

□

If $\Lambda^{*}=id$ is held, then we obtain the following inequality:

$$I^{\left(\Delta \right)}\left(\rho ;id\right)>S\left(\rho \right)$$

It shows that the mutual entropy defined by using the entangled compound state $\Psi_{E,id}^{\left(\Delta \right)}$ with respect to a subset Δ⊂Q$\left(\#\left(\Delta \right)\geq 2\right)$, the initial state ρ and the quantum channel $\Lambda^{*}=id$ greater than the von Neumann entropy $S\left(\rho \right)$ of the initial state ρ.

If the above completely positive channel $\Lambda^{*}$ has orthogonality (i.e., $E_{n}⊥E_{m}\;\left(n\neq m\right)\Rightarrow \;\Lambda^{*}\left(E_{n}\right)⊥\Lambda^{*}\left(E_{m}\right)$) then we have the following theorem.

**Theorem** **19.** For a Schatten decomposition of the initial state ρ, let $\Psi_{E,\Lambda^{*}}^{\left(\Delta \right)}$ be an entangled compound state with respect to a subset Δ⊂Q$\left(\#\left(\Delta \right)\geq 2\right)$ and the CP channel $\Lambda^{*}\left(\rho \right)=\sum_{n}V_{n}\left(\rho \right)V_{n}^{*}$ for any $\rho \in S\left(H_{1}\right)$ with $\sum_{n}V_{n}^{*}V_{n}=I$ and $V_{m}^{*}V_{n}=\delta_{mn}V_{n}^{*}V_{n}$ and orthogonality (i.e., $E_{n}⊥E_{m}\;\left(n\neq m\right)\Rightarrow \;\Lambda^{*}\left(E_{n}\right)⊥\Lambda^{*}\left(E_{m}\right)$). It satisfies two marginal conditions

$$tr_{H_{2}}\Psi_{E,\Lambda^{*}}^{\left(\Delta \right)}=\rho \;\mathrm{and}\;\;tr_{H_{1}}\Psi_{E,\Lambda^{*}}^{\left(\Delta \right)}=\Lambda^{*}\left(\rho \right).$$

*The following inequality is held:*

$$I^{\left(\Delta \right)}\left(\rho ;\Lambda^{*}\right)\geq S\left(\Psi_{E,\Lambda^{*}}^{\left(\Delta \right)},\rho ⊗\Lambda^{*}\left(\rho \right)\right)>S\left(\rho \right)$$

**Proof.**  The relative entropy between $\Psi_{E,\Lambda^{*}}^{\left(\Delta \right)}$ and $\sigma_{0}=\rho ⊗\Lambda^{*}\rho$ is obtained by

$$\begin{array}{cc} & S\left(\Psi_{E,\Lambda^{*}}^{\left(\Delta \right)},\rho ⊗\Lambda^{*}\rho \right)\\ = & tr\Psi_{E,\Lambda^{*}}^{\left(\Delta \right)}\log\Psi_{E,\Lambda^{*}}^{\left(\Delta \right)}-tr\Psi_{E,\Lambda^{*}}^{\left(\Delta \right)}\log\rho ⊗\Lambda^{*}\rho \\ = & S\left(\rho \right)+S\left(\Lambda^{*}\rho \right)\\ & +\sum_{n}\left(\sum_{n_{i}\in \Delta }\lambda_{n_{i}}{∥V_{n}|x_{n_{i}}\rangle ∥}^{2}\right)\log\left(\sum_{n_{j}\in \Delta }\lambda_{n_{j}}{∥V_{n}|x_{n_{j}}\rangle ∥}^{2}\right)\\ & +\sum_{n}\sum_{n_{k}\in Q\backslash \Delta }\lambda_{n_{k}}{∥V_{n}|x_{n_{k}}\rangle ∥}^{2}\log{∥V_{n}|x_{n_{k}}\rangle ∥}^{2}\\ & +\sum_{n_{k}\in Q\backslash \Delta }\lambda_{n_{k}}\log\lambda_{n_{k}}\\ = & S\left(\rho \right)-\sum_{n}\sum_{n_{k}\in \Delta }\lambda_{n_{k}}{∥V_{n}|x_{n_{k}}\rangle ∥}^{2}\log\frac{\lambda_{n_{k}}{∥V_{n}|x_{n_{k}}\rangle ∥}^{2}}{\left(\sum_{n_{j}\in \Delta }\lambda_{n_{j}}{∥V_{n}|x_{n_{j}}\rangle ∥}^{2}\right)}\\ > & S\left(\rho \right) \end{array}$$

Therefore, we obtain the following inequality:

$$I^{\left(\Delta \right)}\left(\rho ;\Lambda^{*}\right)\geq S\left(\Psi_{E,\Lambda^{*}}^{\left(\Delta \right)},\rho ⊗\Lambda^{*}\left(\rho \right)\right)>S\left(\rho \right).$$

□

It shows that the mutual entropy defined by using the entangled compound state $\Psi_{E,\Lambda^{*}}^{\left(\Delta \right)}$ with respect to a subset Δ⊂Q$\left(\#\left(\Delta \right)\geq 2\right)$, the initial state ρ and the quantum channel $\Lambda^{*}$ greater than the von Neumann entropy $S\left(\rho \right)$ of the initial state ρ.

Let $\left\{x_{j}\right\},\left\{y_{j}^{\left(n\right)}\right\}$ be CONS in $H_{1}$ and $H_{2}$. We define a linear map $V_{n}$ from $H_{1}$ to $H_{2}$ by

$$V_{n}|x_{j}\rangle =\sum_{j}\mu_{j}|y_{j}^{\left(n\right)}\rangle .$$

The completely positive channels $\Lambda^{*}$ given by

$$\Lambda^{*}\left(\rho \right)=\sum_{n}V_{n}\left(\rho \right)V_{n}^{*}$$

satisfies

$$\sum_{n}V_{n}^{*}V_{n}=I\;$$

**Theorem** **20.** For a Schatten decomposition of the initial state ρ, let $\Psi_{E,\Lambda^{*}}^{\left(\Delta \right)}$ be an entangled compound state with respect to a subset Δ⊂Q$\left(\#\left(\Delta \right)\geq 2\right)$ and the CP channel $\Lambda^{*}\left(\rho \right)=\sum_{n}V_{n}\left(\rho \right)V_{n}^{*}$ for any $\rho \in S\left(H_{1}\right)$ with $\sum_{n}V_{n}^{*}V_{n}=I$ and $\langle y_{n_{k}}^{\left(n\right)},y_{n_{k}}^{\left(m\right)}\rangle =\delta_{mn}$. It satisfies two marginal conditions

$$tr_{H_{2}}\Psi_{E,\Lambda^{*}}^{\left(\Delta \right)}=\rho \;and\;\;tr_{H_{1}}\Psi_{E,\Lambda^{*}}^{\left(\Delta \right)}=\Lambda^{*}\left(\rho \right).$$

The mutual entropy type measure $I^{\left(\Delta \right)}\left(\rho ;\Lambda^{*}\right)$ increases in proportion to the rise in cardinality $\#\left(\Delta \right).$ It holds the following inequality:

$$I^{\left(\Delta \right)}\left(\rho ;\Lambda^{*}\right)>S\left(\rho \right).$$

**Proof.**  One has

$$\begin{array}{cc} & \Psi_{E,\Lambda^{*}}^{\left(\Delta \right)}\\ = & \sum_{n}\left(id⊗V_{n}\right)\left[\sum_{n_{i}\in \Delta }\sqrt{\lambda_{n_{i}}}|x_{n_{i}}\rangle ⊗|x_{n_{i}}\rangle \right]\\ & \cdot \left[\sum_{n_{j}\in \Delta }\sqrt{\lambda_{n_{j}}}\langle x_{n_{j}}|⊗\langle x_{n_{j}}|\right]\left(I⊗V_{n}^{*}\right)\\ & +\sum_{n}\left(id⊗V_{n}\right)\left[\sum_{n_{k}\in Q\backslash \Delta }\lambda_{n_{k}}E_{n_{k}}⊗E_{n_{k}}\right]\left(I⊗V_{n}^{*}\right)\\ = & \sum_{n}{\left|\mu_{n}\right|}^{2}{∥|\phi_{n}^{\left(\Delta \right)}\rangle ∥}^{2}\frac{|\phi_{n}^{\left(\Delta \right)}\rangle }{∥|\phi_{n}^{\left(\Delta \right)}\rangle ∥}\frac{\langle \phi_{n}^{\left(\Delta \right)}|}{∥|\phi_{n}^{\left(\Delta \right)}\rangle ∥}\\ & +\sum_{n}{\left|\mu_{n}\right|}^{2}\sum_{n_{k}\in Q\backslash \Delta }\lambda_{n_{k}}E_{n_{k}}⊗|y_{n_{k}}^{\left(n\right)}\rangle \langle y_{n_{k}}^{\left(n\right)}|, \end{array}$$

where

$$\begin{array}{ccc} |\phi_{n}^{\left(\Delta \right)}\rangle & = & \sum_{n_{i}\in \Delta }\sqrt{\lambda_{n_{i}}}|x_{n_{i}}\rangle ⊗\mu_{n}|y_{n_{k}}^{\left(n\right)}\rangle ,\\ {∥|\phi_{n}^{\left(\Delta \right)}\rangle ∥}^{2} & = & \sum_{n_{k}\in \Delta }\lambda_{n_{k}} \end{array}$$

Since

$$\begin{array}{ccc} tr\Psi_{E,\Lambda^{*}}^{\left(\Delta \right)}\log\Psi_{E,\Lambda^{*}}^{\left(\Delta \right)} & = & \sum_{n}{\left|\mu_{n}\right|}^{2}{∥|\phi_{n}^{\left(\Delta \right)}\rangle ∥}^{2}\log{\left|\mu_{n}\right|}^{2}{∥|\phi_{n}^{\left(\Delta \right)}\rangle ∥}^{2}\\ & & +\sum_{n}{\left|\mu_{n}\right|}^{2}\sum_{n_{k}\in Q\backslash \Delta }\lambda_{n_{k}}\log{\left|\mu_{n}\right|}^{2}\lambda_{n_{k}} \end{array}$$

under the condition

$$\begin{array}{ccc} \sum_{n}\left(I⊗V_{n}\right)\left(I⊗V_{n}^{*}\right) & = & I⊗I\\ \;\mathrm{and}\;\;\Lambda^{*}\left(\rho \right) & = & \sum_{n}V_{n}\rho V_{n}^{*}\;\mathrm{for}\;\mathrm{any}\;\rho \in S\left(H_{1}\right)\;\\ \mathrm{with}\;\sum_{n}V_{n}^{*}V_{n} & = & I\;\mathrm{and}\;\langle y_{n_{k}}^{\left(n\right)},y_{n_{k}}^{\left(m\right)}\rangle =\delta_{mn} \end{array}$$

$\Psi_{E,\Lambda^{*}}^{\left(\Delta \right)}$ holds two marginal conditions

$$tr_{H_{2}}\Psi_{E,\Lambda^{*}}^{\left(\Delta \right)}=\rho \;\mathrm{and}\;\;tr_{H_{1}}\Psi_{E,\Lambda^{*}}^{\left(\Delta \right)}=\Lambda^{*}\left(\rho \right).$$

The relative entropy between $\Psi_{E,\Lambda^{*}}^{\left(\Delta \right)}$ and $\sigma_{0}=\rho ⊗\Lambda^{*}\rho$ is obtained by

$$\begin{array}{cc} & S\left(\Psi_{E,\Lambda^{*}}^{\left(\Delta \right)},\rho ⊗\Lambda^{*}\rho \right)\\ = & S\left(\rho \right)-\sum_{n_{i}\in \Delta }\lambda_{n_{i}}\log\frac{\lambda_{n_{i}}}{\left(\sum_{n_{j}\in \Delta }\lambda_{n_{j}}\right)} \end{array}$$

Thus, we have the inequality

$$I^{\left(\Delta \right)}\left(\rho ;\Lambda^{*}\right)>S\left(\rho \right).$$

For $\Delta \subset \Delta^{\prime }\subset Q$, one has

$$\begin{array}{cc} & -\sum_{n_{i}\in \Delta }\lambda_{n_{i}}\log\frac{\lambda_{n_{i}}}{\left(\sum_{n_{j}\in \Delta }\lambda_{n_{j}}\right)}\\ \leq & -\sum_{n_{i}\in \Delta }\lambda_{n_{i}}\log\frac{\lambda_{n_{i}}}{\left(\sum_{n_{j}\in \Delta }\lambda_{n_{j}}\right)}\frac{\left(\sum_{n_{j}\in \Delta }\lambda_{n_{j}}\right)}{\left(\sum_{n_{j}\in \Delta^{\prime }}\lambda_{n_{j}}\right)}\\ & -\sum_{n_{\ell }\in \Delta^{\prime }\backslash \Delta }\lambda_{n_{\ell }}\log\frac{\lambda_{n_{\ell }}}{\left(\sum_{n_{j}\in \Delta^{\prime }}\lambda_{n_{j}}\right)}\\ \leq & -\sum_{n_{i}\in \Delta }\lambda_{n_{i}}\log\frac{\lambda_{n_{i}}}{\left(\sum_{n_{j}\in \Delta }\lambda_{n_{j}}\right)}-\sum_{n_{i}\in \Delta }\lambda_{n_{i}}\log\frac{\left(\sum_{n_{j}\in \Delta }\lambda_{n_{j}}\right)}{\left(\sum_{n_{j}\in \Delta^{\prime }}\lambda_{n_{j}}\right)}\\ & -\sum_{n_{\ell }\in \Delta^{\prime }\backslash \Delta }\lambda_{n_{\ell }}\log\frac{\lambda_{n_{\ell }}}{\left(\sum_{n_{j}\in \Delta^{\prime }}\lambda_{n_{j}}\right)} \end{array}$$

Therefore, the mutual entropy type measure $I^{\left(\Delta \right)}\left(\rho ;\Lambda^{*}\right)$ increases in proportion to the rise in cardinality $\#\left(\Delta \right).$ □

If $\#\left(\Delta \right)\leq 1$ is held, then the mutual entropy type measure $I^{\left(1\right)}\left(\rho ;\Lambda^{*}\right)$ is equals to the Ohya mutual entropy taking the von Neumann entropy of the initial state ρ

$$I^{\left(1\right)}\left(\rho ;\Lambda^{*}\right)=I\left(\rho ;\Lambda^{*}\right)=S\left(\rho \right)$$

If Δ=Q is held, then the mutual entropy type measure $I^{\left(Q\right)}\left(\rho ;\Lambda^{*}\right)$ is equals to the Lindblad-Nielsen entropy taking two times of the von Neumann entropy of the initial state ρ

$$I^{\left(Q\right)}\left(\rho ;\Lambda^{*}\right)=I_{LN}\left(\rho ;\Lambda^{*}\right)=2S\left(\rho \right)$$

It shows that the mutual entropy defined by using the entangled compound state $\Psi_{E,id}^{\left(\Delta \right)}$ with respect to a subset Δ⊂Q$\left(\#\left(\Delta \right)\geq 2\right)$, the initial state ρ and the quantum channel $\Lambda^{*}$ greater than the von Neumann entropy $S\left(\rho \right)$ of the initial state ρ. It does not satisfy the fundamental inequalities.

## 8. Conclusions

As is mentioned above, we discuss the quantum mutual entropy type measure by means of the entangled compound state. The mutual entropy type measure at time *t* defined by using the entangled compound state greater than the von Neumann entropy $S\left(\Lambda_{t}^{*}\rho \right)$ of the final state $\Lambda_{t}^{*}\rho$. and the mutual entropy type measure at initial time t=0 greater than the von Neumann entropy $S\left(\rho \right)$ of the initial state ρ. The mutual entropy type measure $I^{\left(\Delta \right)}\left(\rho ;\Lambda^{*}\right)$, which greater than $S\left(\rho \right)$, increases in proportion to the rise in cardinality $\#\left(\Delta \right).$ It does not satisfy the fundamental inequalities. It seems reasonable to suppose that the quantum mutual entropy type measure defined by using the entangled compound state is not useful to discuss the efficiency of information transmission from the initial system to the final system.
